# Enhancing Prednisone-Based Arthritis Therapy with Targeted IL-27 Gene Delivery

**DOI:** 10.3390/bioengineering9060248

**Published:** 2022-06-09

**Authors:** Adriana A. Marin, Richard E. Decker, Shreya Kumar, Zachary Lamantia, Hiroki Yokota, Todd Emrick, Marxa L. Figueiredo

**Affiliations:** 1 Department of Basic Medical Sciences, Purdue University, 625 Harrison St., West Lafayette, IN 47907, USA; alejamarin21@gmail.com (A.A.M.); dr.rdecker16@gmail.com (R.E.D.); kumar308@purdue.edu (S.K.); zlamanti@purdue.edu (Z.L.); 2 Department of Biomedical Engineering, Indiana University Purdue University Indianapolis, Indianapolis, IN 46202, USA; hyokota@iupui.edu; 3 Department of Polymer Science & Engineering, University of Massachusetts, 120 Governors Drive, Amherst, MA 01003, USA; tsemrick@mail.pse.umass.edu; 4 Purdue Center for Cancer Research and Purdue Institute for Drug Discovery, Purdue University, West Lafayette, IN 47907, USA

**Keywords:** targeted Interleukin-27, 27pL, gene delivery, prednisone, rheumatoid arthritis, cytokines

## Abstract

Rheumatoid arthritis (RA) is a chronic autoimmune disease which is characterized primarily by synovial hyperplasia and accumulation of several types of immune infiltrates that promote progressive destruction of the articular structure. Glucocorticoids are often prescribed to treat RA because of their strong anti-inflammatory and immunosuppressive effects. However, their application must be limited to the short-term due to a risk of adverse events. In the present study, we examined the potential combination of low-dose prednisone with gene delivery of an agent of promising and complementary effectiveness in RA, interleukin (IL)-27. IL-27 has been shown to have anti-inflammatory potential, while also acting as an effective bone-normalization agent in prior reports. The present report examined a version of IL-27 targeted at the C-terminus with a short ‘peptide L’ (pepL, LSLITRL) that binds the interleukin 6 receptor α (IL-6Rα) upregulated during inflammation. By focusing on this targeted form, IL-27pepL or 27pL, we examined whether the anti-inflammatory potential of prednisone (at a relatively low dose and short duration) could be further enhanced in the presence of 27pL as a therapy adjuvant. Our results indicate that 27pL represents a novel tool for use as an adjuvant with current therapeutics, such as prednisone, against inflammatory conditions.

## 1. Introduction

Rheumatoid arthritis (RA) is a chronic autoimmune disease which is characterized primarily by synovial hyperplasia in peripheral joints and accumulation of several types of immune infiltrates that promote progressive destruction of joint structure [1]. Affected joints display swelling, erythema, and pain, which may progress to bone erosion and the development of disability [2]. The collagen antibody-induced arthritis (CAIA) mouse model [3] has similar features to human RA, but induces pathology more efficiently relative to another common rodent model of RA, the collagen-induced arthritis (CIA) [4]. In essence, CAIA produces a rapid induction of synchronized and controlled disease progression with histopathological similarities to CIA. CAIA is useful in enabling one to investigate the effector phase of the disease, including synovitis and articular structure alterations. It is induced using a cocktail of antibodies (anti-collagen II) and presents features similar to human RA including pannus formation, leukocytic infiltration, synovitis, and cartilage and bone destruction.

Glucocorticoids are often prescribed to treat RA because of their strong anti-inflammatory and immunosuppressive effects [5]. However, their application must be limited to the short-term due to a risk of adverse events including alterations in bone density once exposure extends past 30 days, for example, in the CIA model of RA [6]. Prednisolone or prednisone is an established treatment in human RA and acts to suppress inflammation and has been shown to prevent bone damage in the CAIA model of RA [6,7]. Recent clinical data support the use of prednisone at low-dose regimens in order to help avoid side effects [8].

In the present study, we wanted to examine the potential combination of prednisone with gene delivery of an agent of promising and complementary effectiveness in RA, interleukin (IL)-27. Besides its potential anti-inflammatory activity [7,9], IL-27 also can serve as an effective bone-normalization agent due to its impact on pro-osteogenic gene changes in both osteoblasts and osteoclasts [10]. IL-27 is a heterodimer of IL-27p28 and EBI3 subunits first identified in 2002 [11], with pleiotropic roles in modulating inflammatory responses [12]. IL-27 is a soluble cytokine that exerts its biological effects by regulating the JAK/STAT signaling pathway via the IL-27RA/IL-6ST receptor pair [13]. A variety of cell and in vivo models indicate that IL-27 can exert multiple regulatory functions in RA through different mechanisms [14].

The current status of IL-27 delivery in arthritis models includes its evaluation in a mouse model of CIA, where it ameliorated inflammation following local delivery via adenovirus [7]. This work utilized native or untargeted IL-27. Two other reports examined intraperitoneal administration of IL-27, which suppressed adjuvant-induced arthritis (AIA) development [15], or examined increased IL-27 production following treatment of CAIA mice with a STAT3 inhibitor, resulting in reduced arthritic inflammation [16]. Recent research on targeted delivery of IL-27 has included liposome encapsulation of the protein, with targeted delivery to arthritic joints achieving significant reduction in the arthritic scores in a rat model of CIA relative to untargeted liposomes [17]. We thus proposed that IL-27 could augment the effect of prednisone in vivo owing to its effects previously described by our group and others, which include anti-inflammatory and anti-angiogenic activity [18], the ability to reduce osteoclastogenic and improve osteoblast maturation [10], and relatively low toxicity [19,20]. Prednisone is a temporary adjunctive anti-inflammatory therapy in RA with similar mechanisms to IL-27 of IL-6 signaling inhibition and several complementary mechanisms, including its ability to inhibit NF-kB through activation of glucocorticoid receptor signaling [21]. We have previously characterized pleiotropic effects for IL-27, such as anti-tumorigenic and pro-osteogenic profiles and also discovered that a targeted form of the cytokine stimulates the expression of luc reporters of STAT-1 and IFNγ in vivo [22]. Our approach is different from the reports described above since we target the cytokine directly and perform gene delivery to achieve IL-27 expression in vivo.

Our prior work examined a first-generation ‘prototype’ of targeted IL-27 we have named IL-27pepL, targeted at the C-terminus with a short ‘peptide L’ (pepL, LSLITRL). pepL binds the interleukin 6 receptor α (IL-6Rα) upregulated in tumor cells [23] and is able to further inhibit IL-6 signaling via enforcing a STAT1-dominant signaling pattern [18,24]. Thus, by focusing on the targeted form of IL-27pepL (27pL), we proposed to examine whether the anti-inflammatory potential of prednisone (at a relatively low dose and short duration) could be further enhanced in the presence of 27pL as a therapy adjuvant.

In this report, 27pL can also target the arthritic joint by virtue of the IL-6Rα expression in immune and bone cells. Here we have generated further enhancements of the IL-27 molecule, containing two motifs of a flexible linker at the C-term, where structural modeling suggested this form of the molecule could enable better accessibility of the peptide ligand for IL-6Rα inhibition. We also made improvements in the vector for enhancing 27pL expression relative to previous utilized versions. Overall, this new vector and IL-27 targeted molecule, 27pL, can be used as a novel adjuvant with current therapeutics, enabling their use at lower, potentially less toxic doses, such as low-dose prednisone against inflammatory conditions.

## 2. Materials and Methods

### 2.1. Vectors and Transgene Detection

Targeted IL-27 vectors were produced using standard cloning, utilizing digestion and ligation methods. A duplex encoding two Gly4Ser sequences as a linker (2xG4S) and the pepL sequence (LSLITRL) was synthesized by IDT (Integrated DNA Technologies, Coralville, IA, USA) to have 5′ and 3′ ‘sticky ends’ compatible with *Xcm*I and *Nhe*I, respectively. This duplex was ligated into the pORF9 (plasmid open reading frame 9) backbone (InvivoGen, San Diego, CA), which had been linearized with *Xcm*I and *Nhe*I, to create a final construct (pORF9-IL-27pL or pORF27pL) encoding EBI3-p28-(2xG4S)-pepL. The non-specific peptide control, EDLGREK, was produced in a similar manner. All vectors were prepared using GeneJET plasmid endofree megaprep kits for in vivo work (Thermo Fisher; Waltham, MA, USA). Real-time quantitative qPCR was utilized to detect expression of the transgene by amplification of the boundary between the EBI3 and IL27p28 cDNAs in the hybrid vectors, using F: 5′-GCCTCCCTGGGCAAGTAGAA-3′ and R: 5′-CTTGAAGGCTCAGGGGGCT-3′ primers. Reverse-transcription was performed for 0.5 µg of each RNA sample using amfiRivert Platinum cDNA Synthesis Master Mix (GenDEPOT, Katy, TX, USA). RT-qPCR reactions contained 1 µL of cDNA template, 2X SYBR Green Master Mix (KAPA Biosystems, Wilmington, MA, USA), and 1 µM of forward and reverse primers for target genes or beta-actin as housekeeping control (IDT). RT-qPCR was performed in a Viia 7 Real-time PCR System (Applied Biosystems, Waltham, MA, USA) using the following conditions 95 °C for 3 min, 40 cycles of 95 °C for 3 s, 60 °C for 30 s and 72 °C for 19 s. Data acquisition was performed with Quant Studio 3 software (Applied Biosystems).

### 2.2. Chemicals and Reagents

For the study, ArthritoMab^TM^ Arthritis Inducing Antibody Cocktail (CIA-MAB-50) and LPS (Escherichia coli strain 055: B5; MDLPS.5) were purchased from MD Biosciences Bioproducts, LLC (Oakdale, MN, USA). Prednisone (P6254-5G) was procured from Sigma–Aldrich (St. Louis, MO, USA). OsteoSense 750X (NEV10053EX) was purchased from PerkinElmer Health Sciences Inc. (Hebron, KY, USA).

### 2.3. Experimental Animals & Sonodelivery of Plasmid DNA Expressing IL-27

Female Balb/c mice (20–30 g) were obtained from Envigo and kept in rooms with controlled temperature and humidity and a 12 h light/dark cycle. For therapy studies, *n* = 5/group were utilized, in duplicate experiments for a total of *n* = 11–12 per therapeutic group. For imaging assessments, *n* = 3 were used per group. The animals were supplied with standard pellet diet and sterile filtered water ad libitum. Mice were handled in accordance with the approved study protocol no. 1508001279 by the Purdue Animal Care and Use Committee (PACUC), an institutional review board for animal research. Plasmid DNA (pDNA) expressing EBI3-IL-27p28.pepL (pORF-27pL) or empty vector (pORF-0) were delivered complexed with the comb-like polymer RL638, containing a reverse nuclear localization signal-phosphocholine(rNLS-PC) composition. The rNLS used was VKRKKKP, appended onto a poly(cyclooctene) backbone and the polymer contained 14 mol% PC zwitterions to improve biocompatibility, synthesized using methods as per [25]. pDNA complexes were delivered to the muscle (I.M) of each hind leg on the day 2 of the experiment. The sonodelivery conditions were 60 s; 1 MHZ; 20% duty, 3 W/cm^2^ using the 10 mm Sonopore NepaGene KTAC4000 probe (Bulldog Bio, Portsmouth, NH, USA) and 30% sonovue Bracco microbubbles (SV-25, Bulldog Bio), conditions previously reported by our group [22].

### 2.4. Induction of Arthritis in Animals

The collagen antibody-induced arthritis (CAIA) model was induced according to the method described in [3]. Briefly, Balb/c mice were injected intraperitoneally (I.P.) using a cocktail of 5 monoclonal antibodies anti-type II collagen (1.5 mg in 1x Dulbecco’s Phosphate Buffered Saline (1xDPBS, 14190144, ThermoFisher)/mouse; day-0) followed by sonodelivery of plasmid DNA expressing IL-27pL (pORF27pL) or empty vector was delivered on the day-2. I.P. injection of 50 μg of lipopolysaccharide (LPS from Escherichia coli strain 055B5; in a sterile normal saline) was performed on day 3. On day 4, disease-induced animals were selected and randomized into different groups for treatments (*n* = 5 per group): (1) no CAIA control (NC) (0.1% DMSO vehicle via oral gavage); (2) CAIA (pORF-0 intramuscular (I.M.) once (day 2) + 0.1% DMSO); (3) CAIA + prednisone (P) + pORF-27pL (10 mg/kg of P in 0.1% DMSO via oral gavage; pORF-27pL (I.M.); (4) CAIA + P (10 mg/kg); pORF-0 (I.M.), and (5) CAIA + pORF-27pL (0.1% DMSO; pORF-27pL (I.M.). The vehicle or P treatments were initiated on day 4 and continued for 7 days.

### 2.5. Assessment of Arthritis Severity

The severity of arthritis in each mouse paw was scored for all days in a blinded manner, according to the method of Khachigian [3] on a scale of 0–4, as follows: 0 = normal; 1 = mild redness, slight swelling of ankle or wrist; 2 = moderate swelling of ankle or wrist; 3 = severe swelling, including some digits, ankle and foot; 4 = maximally inflamed. The total maximum score for each mouse was 16 [3]. The anti-arthritic activity (%) was calculated for each animal using the following formula: ((Mean arthritis score of disease control animals—arthritis score of each test animal)/Mean arthritis score of disease control animals) × 100 [26].

### 2.6. Measurement of Paw and Ankle Joint Thickness

Mouse paw and ankle thickness were measured in a blinded manner using digital vernier calipers from day 0 and consecutively until day 10 post-induction.

### 2.7. Assessment of Bone Damage by Imaging Using an OsteoSense 750X Probe

NIR fluorescent bisphosphonate agent was used to bind, with high affinity, to hydroxyapatite (HA) that enables imaging of areas undergoing bone mineral changes such as those occurring during bone remodeling. On day 5 post-induction, mice from all groups were injected intraperitoneally with 4 nmol/100 µL 1x DPBS of OsteoSense750x per mouse (*n* = 3 per group). On days 6, 8, and 10 the fluorescence signal from animal limbs were monitored through an Ami HTX optical imaging system (Spectral Instruments Imaging, Tucson, AZ, USA). The exposure time ranged from 60 to 120 s depending on the signal strength. The mean values of radiation (photons/s/cm^2^/sr) were calculated for the regions of interest (ROI) using AURA Image software for all experimental animals. Background values were obtained from the NC group of mice injected with the probe and were subtracted prior to the analysis.

### 2.8. Histopathological Examination

On day 10 post-induction, the animals were euthanized, and their hind limbs were harvested and fixed in 10% buffered-neutral formalin for 48 h, decalcified in 10% formic acid for four days, embedded in paraffin, sectioned at a thickness of 3–5 μm and stained with hematoxylin and eosin (HE) for general evaluation or Safranin O for assessment of cartilage degradation (*n* = 3 per group). Blinded examination of histological slides was performed to minimize bias. The severity of microscopic arthritic changes (synovial hyperplasia, synovial vascularity, infiltration of inflammatory cells, pannus formation, cartilage erosion, and bone erosion) were evaluated in HE-stained slides using the following grades: 0 = no abnormality detected; 1 = minimal (<1%); 2 = mild (1–25%); 3 = moderate (26–50%); 4 = marked (51–75%); 5 = severe (76–100%). Similarly, the severity of the cartilage degradation was scored as 0 = no apparent change; 1 = superficial fibrillation of articular cartilage; 2 = defects limited to uncalcified cartilage; 3 = defects extending into calcified cartilage; 4 = exposure of subchondral bone at the articular surface. The scoring for the knee and ankle joints was recorded separately [26,27]. Images of the histological slides (HE and Safranin O) were captured using Olympus microscope camera and processed by Olympus cellSens imaging software.

### 2.9. Multiplex Cytokine Assay

On day 10 post-induction, the animals were euthanized, and blood samples were collected and kept for 15 min at 4 °C until its coagulation. Samples were centrifuged at 10,000× *g* for 5 min and the supernatant was transferred into new tubes and stored at −80 °C until analysis. Cytokines such as, G-CSF, IFN-γ, IL-1β, IL-6, IL-10, IL-13, TNF-α, and RANTES (CCL5) were analyzed through the Mouse Cytokine/Chemokine Discovery Assay by Eve technologies (Calgary, AB, Canada).

### 2.10. Statistical Analysis

The data are expressed as the mean ± standard error of the mean (SEM) for each experiment. For comparison of means of more than two treatments, a one-way analysis of variance (ANOVA) followed by Tukey‘s multiple comparison posttest was used to calculate the statistical difference between all groups. Mann–Whitney‘s nonparametric test was used to calculate the statistical differences between two groups. *P* values of <0.05 were considered statistically significant. The analyses were performed using GraphPad PRISM software version 7.

## 3. Results

### 3.1. Generation of Targeted IL-27 Construct and Testing in Cells

**Figure 1:**
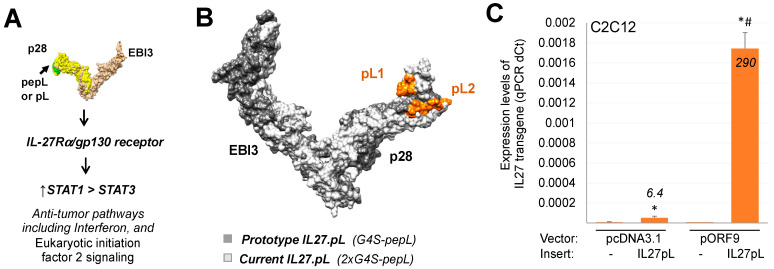
Overview of the IL-27pepL features. (**A**) Schematics of IL-27pepL functions. (**B**) Modeling and alignment of the prototype IL-27pepL containing 1 Gly4Ser (G4S) linker or current version with 2xG4S linker with the pepL in the pL1 (prototype) or pL2 (current) conformations, using iTASSER and Chimera. (**C**) Expression levels by qPCR of the IL-27pL transgene in C2C12 muscle cells comparing the traditional vector pcDNA3.1 and the new pORF9 vectors (lacking an insert (−) or with the IL-27pL insert) generated in this study. *, *p* < 0.05 relative to vector lacking insert, and #, *p* < 0.05 relative to the pcDNA3.1-IL-27pL group.

Prior work from our group indicated promise for IL-27 in modulating pathways [28] that could aid in arthritis treatment. Also, several recent projects from our group had indicated that IL-27 could be modified at the C-terminus with a short peptide that enhanced its therapeutic efficacy relative to wild-type or native IL-27 [22,29,30]. This peptide consisted of a tumor-targeting and IL-6Rα-antagonistic motif, ‘pepL’ (LSLITRL) [23] (Figure 1A). In this work, we sought to enhance the construct utilized by placing the IL-27pepL (27pL) in a pORF9 backbone, hypothesized to have a promoter (EF1α/HTLV hybrid) of reported superior expression strength and stability in cells and in vivo [31,32,33] relative to our previously reported CMV promoter in the pcDNA3.1 backbone. Another improvement made to this vector was the addition of two Gly4Ser linkers for better positioning of the pepL at the C-terminus from the EBI3-IL27p28 fusion protein, proposed by modeling to enable better accessibility of the peptide ligand for IL-6Rα inhibition. For example, the new pepL2 ligand conformation is more fully accessible for binding relative to the former pepL1 configuration (Figure 1B).

We assessed the levels of transgene using quantitative real-time PCR in C2C12 muscle cells transfected with either vector, and detected higher levels of 27pL from the pORF9 backbone relative to a pcDNA3.1 backbone (Figure 1C). This new-generation pORF9-27pL (pORF27pL) vector was then employed as a monotherapy or as a dual therapy along with low-dose prednisone (P) in a mouse model of RA.

### 3.2. 27pL and Prednisone (P) Therapeutic Application in the CAIA Model of Arthritis

To examine the impact of 27pL on augmenting P therapy, we examined their performance as mono- or dual therapies in the CAIA mouse model of RA. For the gene delivery protocol, we utilized previously developed strategies of sonoporation-mediated gene delivery (sonodelivery), optimized using reporter gene plasmids, finding that the best approach utilized plasmid DNA complexed with biocompatible, cationic polymers [22,25], such as the one employed in the current work. For the P dosage and route of administration [2,34], we referred to the literature on previous work in the CAIA model.

**Figure 2:**
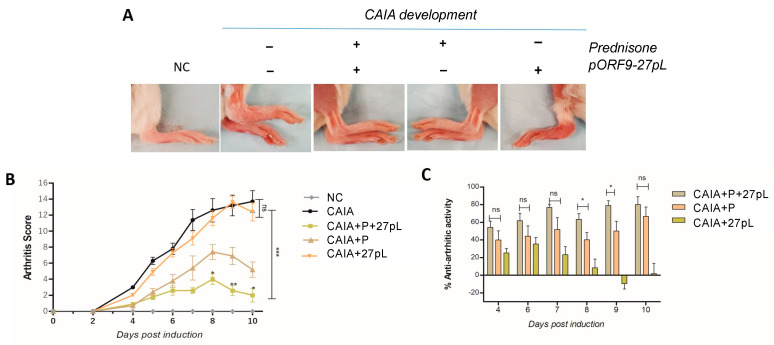
Modulation of arthritis severity by monotherapies or dual therapy of prednisone (P) and pORF9-IL27pL (27pL). (**A**) Morphological changes of digits, foot, and ankle edema in collagen antibody-induced arthritis (CAIA) mice with and without treatments and NC (no CAIA control). (**B**) Arthritis score showed an increase in the CAIA animals indicating the onset of RA. Treatment of the CAIA mice with P significantly reduced the arthritis score indicating a reduction in arthritis severity. Combination with pORF9-IL27pL (27pL) significantly improves P’s effect on day 8, 9, and 10 post-induction. 27pL treatment by itself was not able to prevent the onset of RA. (**C**) Anti-arthritic activity analysis showed efficacy for P monotherapy and P + 27pL combination on day 8 and 9. Results represent mean ± SEM. This figure is the combination of two independent experiments (*n* = 11–12). A one-way analysis of variance (ANOVA) followed by Tukey’s multiple comparison *t*-test was used to calculate the statistical difference. The Mann–Whitney non-parametric test was used to calculate the statistical difference between P and P + 27pL (* *p* ≤ 0.05; ** *p* ≤ 0.01, *** *p* ≤ 0.001, with ns, not significant).

We assessed the effect of therapies in the CAIA model and evaluated a series of parameters. In Figure 2A, although changes observed were subtle for the pORF9-27pL-containing groups, the morphological changes in ankles and paws were assessed to produce quantifiable arthritis scores (Figure 2B), as detailed in materials and methods. CAIA animals showed, as expected, higher arthritic scores due to swelling of the ankles and digits (Figure 2A). Treatment of the CAIA mice with P significantly reduced the arthritis score, indicating a reduction in arthritis severity as a trend. When combined with the 27pL vector, the effect of P was enhanced, yielding a significant improvement on the arthritis scores on days 8, 9, and 10 post-induction (Figure 2B). 27pL as a monotherapy was unable to prevent the onset of RA. A different analysis evaluating the anti-arthritic activity of each monotherapy or dual therapy based on the arthritis score [26] showed efficacy for P as a monotherapy, and confirmed that the dual therapy of P + 27pL further enhanced the anti-arthritic activity on days 8 and 9 (Figure 2C).

**Figure 3:**
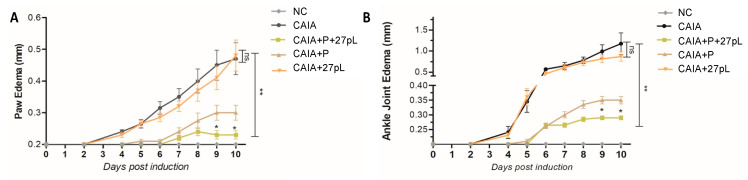
Prenidsone reduced ankle joint and paw edema and this effect was enhanced by 27pL. (**A**) CAIA animals developed paw edema, but treatment with Prenidsone (P) significanly reduced this phenotype. Dual therapy with P + 27pL further improved paw edema on days 9 and 10 post-induction. (**B**) Increase in ankle edema is also typically observed in CAIA animals, yet P significantly reduced this phenotype, with dual P + 27pL further improving ankle edema on days 9 and 10 post-induction. Results represent mean ± SEM. This figure is the combination of two independent experiments. A one-way analysis of variance (ANOVA) followed by Tukey’s multiple comparison *t*-test was used to calculate the statistical difference. The Mann–Whitney non-parametric test was used to calculate the statistical difference between P and P + 27pL (* *p* ≤ 0.05; ** *p* ≤ 0.01), with ns, not significant.

Since CAIA animals typically develop paw and joint edema, i.e., relevant features of RA in humans, we sought to begin our analysis of the impact of P and 27pL by examining this phenotype. Relative to the no CAIA (NC) control, treatment with P significantly reduced edema both in the front paws (Figure 3A) and ankles (Figure 3B). Dual therapy with P + 27pL further improved edema on days 8 through 10 post-induction (Figure 3). These data suggested we could also detect improvements in these joints via histopathological analyses.

**Figure 4:**
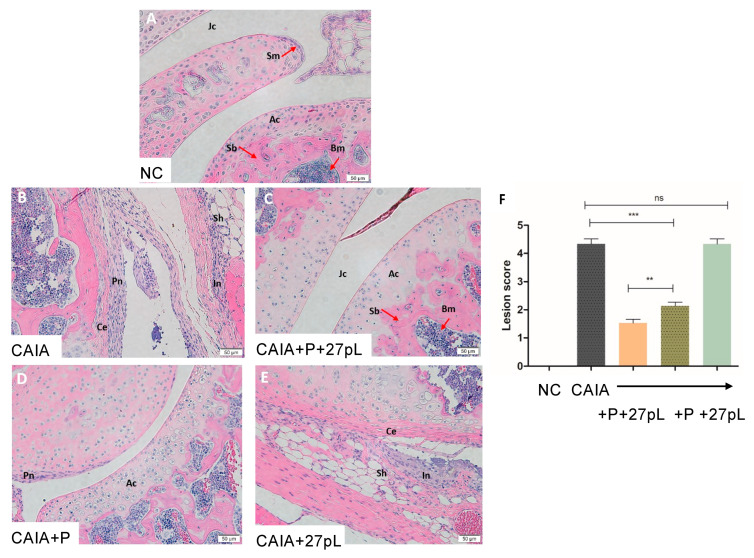
Histopathological analysis of the knee joint. (**A**) No CAIA control (NC, -ctrl) knee showing articular cartilage (Ac), synovial membrane (Sm), subchondral bone (Sb), bone marrow cells (Bm), and the joint cavity (Jc). (**B**) CAIA mice (+ctrl) showed hyperplastic synovium (Sh) with increased Pannus formation (Pn) and Cartilage erosion (Ce). (**C**) Treatment of the CAIA mice with prednisone P + 27pL showed a lack of hyperplastic synovium, inflammation, and pannus formation. The joint cavity (Jc), articular cartilage (Ac), Subchondral bone (Sb) and Bone marrow (Bm) were similar to NC. (**D**) Treatment of CAIA mice with P showed only a low decrease in Pannus formation (Pn), and articular cartilage (Ac) integrity was preserved. (**E**) CAIA mice treated with 27pL showed hyperplastic synovium (Sh), inflammation (In), pannus formation (not shown), and cartilage erosion (Ce). (**F**) Total lesion scores indicated increased inflammatory lesions in the CAIA mice. Treatment with P reduced the lesion score of knee joints, and 27pL expression potentiated the effect of P. Results represent mean ± SEM. A one-way analysis of variance (ANOVA) followed by Tukey’s multiple comparison *t*-test was used to calculate the statistical difference. The Mann–Whitney non-parametric test was used to calculate the statistical difference between P and P + 27pL (** *p* ≤ 0.01; *** *p* < 0.001), with ns, not significant.

Histopathological analyses of the knee joint stained with hematoxylin and eosin (HE) showed that, relative to the untreated control, the treatment with P + 27pL was the most effective in reducing lesion scores, as illustrated in Figure 4. For example, mice treated with P + 27pL displayed reductions in hyperplastic synovium, pannus formation, inflammation features, and other features, including reduced cartilage erosion. P alone had residual pannus but improved cartilage erosion (Figure 4D), whereas features of synovial hyperplasia, inflammation, and cartilage erosion were not significantly improved relative to the CAIA control for 27pL treated animals (Figure 4E). The lesion scores (combined) are presented in Figure 4F.

**Figure 5:**
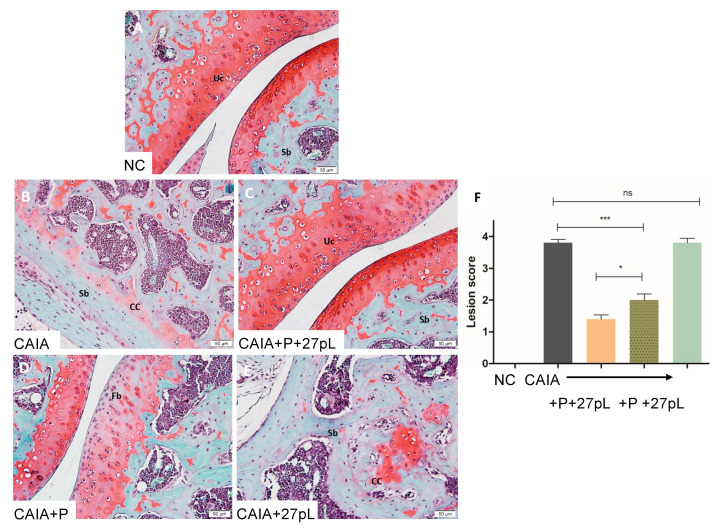
Histopathological analysis of articular cartilage erosion of knee joint. (**A**) Histological analysis of no CAIA (NC) control animal knee joint stained with safranin ‘O’ showed normal uncalcified cartilage (Uc), and the subchondral bone (Sb). (**B**) Knee joint in CAIA mice (+ctrl) showed cartilage degradation extending up to the Sb and the presence of calcified cartilage (CC). (**C**) Treatment of the CAIA mice with P + 27pL showed Uc, and an absence of cartilage degradation or CC. (**D**) Treatment of the CAIA mice with P showed superficial fibrillation (Fb) of the articular cartilage. (**E**) Treatment of CAIA mice with 27pL showed degradation of cartilage and calcified cartilage and limited therapeutic effectiveness, as evidenced by Sb and CC abnormal morphology. (**F**) Total lesion score measurement indicated increased inflammatory lesions in the CAIA mice. Treatment with P showed a significant reduction in the lesion score of cartilage degradation and 27pL potentiated this effect. Results represent mean ± SEM. A one-way analysis of variance (ANOVA) followed by Tukey’s multiple comparison t-test was used to calculate the statistical difference. The Mann–Whitney non-parametric test was used to calculate the statistical difference between P and P + 27pL (* *p* ≤ 0.05; *** *p* < 0.001, with ns, not significant).).

Next, we also examined the extent of articular erosion in the knee joint by specialized staining utilizing Safranin O, a cationic dye that stains for proteoglycans and glycosaminoglycans [35] to help assess the integrity of cartilage in the CAIA joints. Relative to the untreated control (Figure 5A), CAIA mice showed extensive cartilage degradation (Figure 5B), with an almost complete loss of red Safranin O staining of normal uncalcified (Uc) cartilage. When treated with P + 27pL, knee joints displayed a virtually normal morphology, lacking both cartilage degradation and calcifications within the articular cartilage (Figure 5C). P alone could not prevent cartilage fibrillation changes (Figure 5D), and 27pL alone could not prevent cartilage degradation nor the presence of calcified cartilage (Figure 5E), with scores being undetectable from CAIA (+ctrl). The combination of P + 27pL significantly improved the lesion score (Figure 5F) relative to the CAIA control or P alone.

Supplementary Figures S1 and S2 show similar trends and observations made for the ankles of CAIA-treated mice treated with P alone or with 27pL. Thus, the CAIA model is useful for determining changes in both knee and ankle joints of mice.

**Figure 6:**
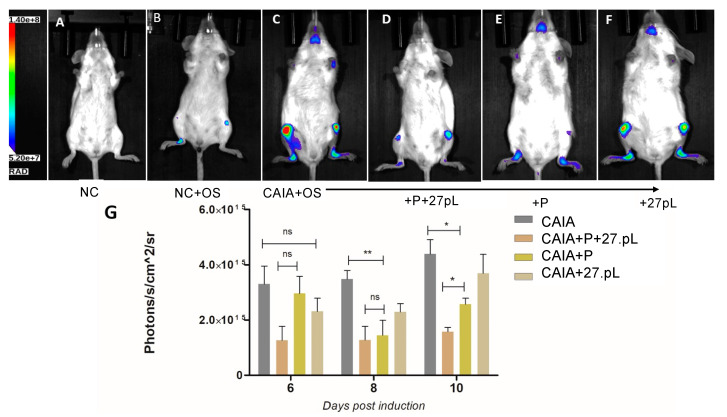
Quantification of bone damage or repair by Osteosense probe fluorescence bioimaging. (**A**) No CAIA (NC) control showing an absence of Osteosense fluorescence. (**B**) NC with an injection of Osteosense showing the basal fluorescence from probe. (**C**) CAIA animals showed extensive damage or changes in bone in the ankle and knee joints, indicating the onset of RA. (**D**) Treatment of CAIA animals with P + 27pL showed significant reduction of bone damage. (**E**) Treatment of CAIA with P showed a reduction in bone damage. (**F**) Treatment of CAIA with 27pL showed no reduction of damage and bone repair. (**G**) Quantification of Osteosense fluorescence on day 6, 8, and 10 post-induction. Treatment with P significantly reduced the bone damage on day 8, and 27pL expression improved its effect on day 10 post-induction. Background (correction) values from the NC + OS group were subtracted in these analyses. Results represent mean ± SEM. A one-way analysis of variance (ANOVA) followed by Tukey’s multiple comparison t-test was used to calculate the statistical difference. The Mann–Whitney non-parametric test was used to calculate the statistical difference between P and P + 27pL (* *p* ≤ 0.05; ** *p* ≤ 0.01), with ns, not significant.

We further examined the extent of damage or repair of bone by utilizing molecular imaging bisphosphonate-based probes (pamidronate; Osteosense, PerkinElmer) for longitudinal imaging of bone metabolism. Figure 6 shows that P + 27pL and P alone (Figure 6D,E) were the most effective in reducing Osteosense signals in knee and ankle joints relative to the CAIA control animals (an example in Figure 6C). The 27pL treatment decreased the Osteosense signals on days 6 and 8, but these trends did not reach statistical significance (Figure 6G). The only group that consistently maintained both a trend in reducing Osteosense signals and statistical significance was the P + 27pL group (Figure 6G).

**Figure 7:**
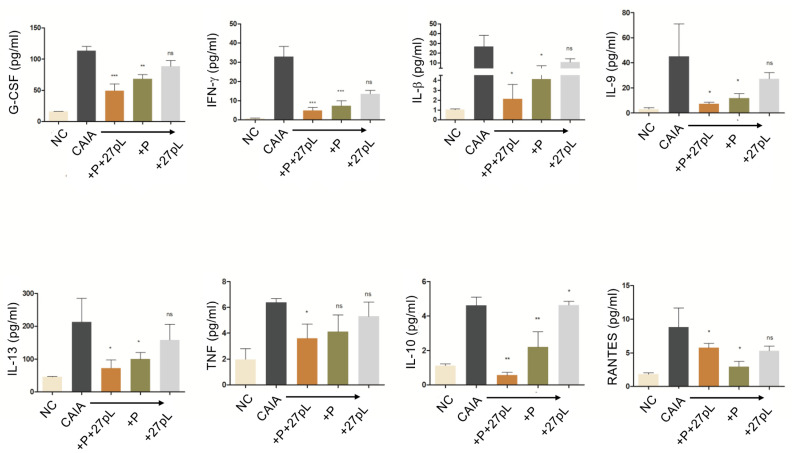
Cytokine quantification in the serum of the no CAIA (NC, − control), CAIA mice (+ control), P-treated, and 27pL-treated CAIA mice using a multiplex cytokine assay. On day 10 post-induction, all animals were euthanized, and the serum was obtained for analysis. NC displayed the basal expression levels reported here. CAIA mice had a significant increase in all cytokines produced. P + 27pL markedly decreased the levels of IL-10, IL-6, TNF-α, IFN-γ, IL-13, IL-1β, G-CSF, and RANTES in the serum of CAIA mice. Results represent mean ± SEM. This figure is the combination of two different experiments. A one-way analysis of variance (ANOVA) followed by Tukey’s multiple comparison *t*-test was used to calculate the statistical difference (* *p* ≤ 0.05; ** *p* ≤ 0.01; *** *p* ≤ 0.001), with ns, not significant.

The expression of several cytokines was assessed in samples collected from mouse serum at the endpoint to better understand the potential mechanisms underlying the higher effectiveness of the P + 27pL combination therapy against the CAIA-induced RA model. Several cytokines may underlie the efficacy of the P + 27pL treatment, markedly decreasing the levels of IL-10, IL-6, TNF-α, IFN-γ, IL-13, IL-1β, G-CSF, and RANTES in the serum of CAIA mice. P alone had similar trends but at a lower magnitude relative to the P + 27pL combination treatment (Figure 7).

**Figure 8:**
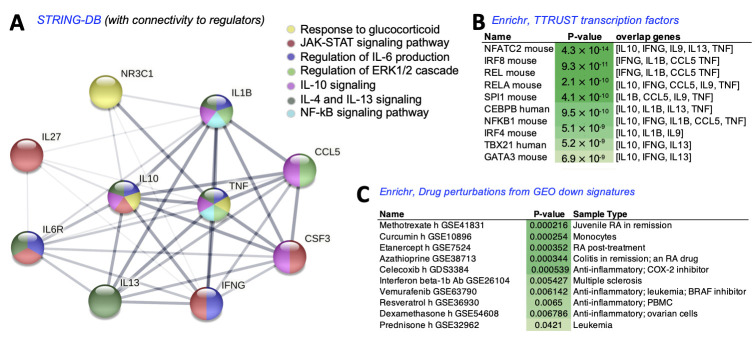
Pathway analysis of the cytokine networks impacted by P + pORF-27pL. (**A**) Protein–protein interaction network from STRING-DB to visualize connections between pathways differentially modulated by P + 27pL relative to monotherapies; (**B**) Enrichr analysis using a TTRUST transcription factors database, and (**C**) Enrichr analysis using the signatures from drug perturbations from GEO down dataset.

A pathway analysis was conducted utilizing *String-DB* [36], focusing on the cytokines significantly altered by the P + 27pL combination relative to the monotherapies. For this analysis, we also input the three candidate regulators from our therapeutics (NR3C1, IL-27, and the pL target, IL6RA) in order to visualize the potential protein-protein network connectivity. This analysis suggested that P, via its NR3C1 mediator, can potentially impact IL-10 and TNF signaling via the Glucocorticoid pathway (yellow nodes, Figure 8A). IL-27 can potentially impact IL-10, IL-6R, IL-13, IFNG, IL-9, and CSF3 via the JAK-STAT pathway (red nodes, Figure 8A). pepL is likely reinforcing the JAK-STAT pathway through IL6R inhibition [23], as well as potentially impacting IL-6 production, IL-4/-13 signaling, and IFNG signaling. Many crosstalk pathways are likely contributing to signaling through several molecules. It seems that the most downstream molecules impacted by both prednisone and IL-27/pepL are IL-10 and CSF3. CCL5, a molecule that displayed a different pattern of expression (higher in P + 27pL relative to P alone), has a network suggestive of regulation by several pathways but not necessarily directly from P or 27pL. These included the regulation of ERK1/2 cascade and IL-10 signaling. A couple of central nodes are potentially regulated by the NFkB signaling pathway. An Enrichr [37,38] analysis using the *TTRUST transcription factors database* ranked potential regulators of the P + 27pL cytokine response by the enriched −log10(p) values. These included NFATC2 and other REL/NFkB regulators, and IRF/TBX21 regulators, related to JAK-STAT signaling (Figure 8B). An Enrichr analysis using the *drug perturbations from GEO down signatures* indicated drugs with similar signatures and included several conditions relating to reduced inflammation and RA, such as methotrexate, curcumin, resveratrol, celecoxib (which include JAK-STAT3 inhibition among its mechanisms [39,40,41,42]), and other anti-inflammatory drugs utilizing diverse mechanisms such as anti-IFNβ1, the TNF inhibitor etanercept, and the BRAF inhibitor vemurafenib (Figure 8C).

## 4. Discussion

In the present work, we examined the potential for gene delivery of a targeted form of IL-27 (IL27.pepL or 27pL) in enhancing the anti-inflammatory effects of prednisone in vivo. Other earlier versions of the cytokine, summarized in Figure 1, have been described recently from our lab, with anti-tumor effects both in cells [30] and in vivo [22,43]. The continued optimization of the cytokine and the delivery vectors has led us to the current version, containing three linkers between the EBI3-IL27p28 and the pepL. The current vector enables high-level expression relative to a former plasmid backbone and thus more promising for in vivo expression. However, despite these improvements, our results in the present work indicated that the cytokine alone did not display the effect on CAIA joints that had been expected based on a report using the CIA model [7], which had indicated a reduction in inflammatory lesions with an adenovirus. There are two possibilities that can help explain this difference: the CIA model develops with slower kinetics of lesion development, and viral vectors can usually deliver genes with a higher efficiency relative to nonviral vectors [44].

Interestingly, although monotherapy was not effective in reducing various imaging or histopathology CAIA phenotypes, there were sometimes trends towards reduced Osteosense signals, at least on days 6 and 8, potentially earlier changes relative to prednisone alone. This is interesting because this is likely the timepoint where the vector peaks following gene delivery to muscle by the strategy we utilized, sonoporation [25]. The networks connecting the cytokines modified as a result of the combination of prednisone and 27pL lead to interesting potential mechanisms of activity. The pathways connected at reduced proinflammatory signaling but this was counterintuitive in the sense that IL10 pathways were reduced. IL-27 is typically thought to mediate anti-inflammatory activity in part through the induction of IL-10 by CD4 T cells via a STAT1/3 dependent pathway [9]. As recently shown for TLR4 induced macrophages, IL-10 can play different roles, either restraining (via IFNβ) or resolving (via TNF) activation of inflammatory responses [45], and either IL-10 is not the main mechanism of anti-inflammatory activity of P + 27pL, or it does not persist at the endpoint. The major anti-inflammatory mechanism at the endpoint might be rather the downregulation in JAK-STAT, IFNG, and CSF3. The analyses that examined potential regulators included NFATC2 and other REL/NFkB regulators, and IRF/TBX21 regulators, related to JAK-STAT signaling. It would appear that P and pL can cooperate therapeutically by modulating different pathways such as NFkB and JAK-STAT. An Enrichr analysis using the *drug perturbations from GEO down signatures* indicated drugs with similar signatures and included several conditions relating to reduced inflammation and RA, such as methotrexate, curcumin, resveratrol, celecoxib, suggesting inhibition of JAK-STAT3 may be a particular way in which pathways modulated by these drugs (or P) can interact with IL-27. Other anti-inflammatory drugs utilized diverse mechanisms such as anti-IFNβ1, the TNF inhibitor etanercept, and the BRAF inhibitor vemurafenib. Potentially, inhibition of the TNF or IFNβ pathways could help augment the efficacy of 27pL alone and could be alternatives to the use of low-dose prednisone. One key limitation of these analyses is that we observed similar ranges in the cytokine assay between P only and 27pL + P, even though these were statistically different. Thus, future analyses should prioritize those cytokines with the most distinct results between P and 27pL + P (i.e., the IL-10-CCL5-IFNγ connection) for continued refinement and development of this combination therapy.

Future studies could examine higher-expression vectors, as well as those optimized towards CpG free content, to help prevent any vector silencing typically reported in vivo following delivery (reviewed in [44]). Also, 27pL could augment the therapeutic efficacy of prednisone in all phenotypes examined, which is promising for the use of this combination therapy. It appears that the IL-27 modality (or C-terminal modified 27pL) will be best utilized in a combination setting, since as a monotherapy, the effectiveness was limited in its current version. The pleiotropic effects of IL-27 in modulating inflammatory responses indeed make it challenging to determine when to optimally use this therapeutic [14]. For example, IL-27 inhibits ectopic-like structure (ELS) formation and CD4 Th2 and Th17 pathogenic helper cells, as well as osteoclastic differentiation in RA, all of which can contribute to alleviating RA. However, IL-27 also can promote Th1 cell differentiation, which may worsen synovitis in RA. IL-27 can act on synovial fibroblasts and Treg, but the functions remain unclear. Thus, our work supports the literature that reports that IL-27 is safe and can be beneficial to treating RA, yet that it should be utilized in the context of a dual therapy, for example, with prednisone.

A final comment is that the utility of gene therapies such as 27pL may require finding the optimal timing and dosing for higher efficacy. Regarding timing, this was illustrated recently in an influenza model, where IL-27 plays an important role in limiting destructive inflammation (protective), particularly in the resolving (late) phase of infection. In this model, IL-27 restricted innate cell recruitment without affecting the T cell response in the local tissues, controlling immunopathology locally without impact on the host defense [46]. The timing could be important to maximizing cytokine therapeutic activity, as shown in a rat model where IL-27 could control the main mediators of inflammation and IFNγ enhanced IL-27 secretion, revealing a cooperative interplay between the cytokines [15]. Regarding dosing, future studies should prioritize examining a range of plasmid concentrations delivered intramuscularly, as well as a range of prednisone concentrations, in order to help refine the combination therapy. Additional improvements in the gene delivery aspect should also continue to be achieved by the designing of improved long-term expression vectors for gene delivery [44].

The present work initiated a road to discovery of new targeted IL-27 molecules that may be able to enhance low-dose RA therapies and may be applicable to other arthritis types. Our group is currently pursuing different ways to also target the IL-27 in other current ongoing projects, which may enhance arthritis-related applications, as well as expand the therapeutic applications for other inflammatory conditions such as those related to infectious or respiratory-distress diseases. The future of RA therapy likely will draw from multiple fields of research, potentially combining strategies such as targeted cytokines and anti-inflammatory drugs, as described here, with other approaches such as regenerative medicine and cellular therapy strategies. Cell therapies may include, for example, mesenchymal stem/stromal cells for delivering cytokines, viruses, or drugs. The ultimate goal for RA therapy will be to develop single or combinatorial interventions that can greatly reduce the side effects seen with systemically-applied therapies. As our understanding grows, RA therapies will continue to be developed that are more precise, with individualized or targeted features, and potentially enable their optimized efficacy while mitigating their potential side effects.

## Data Availability

Data is available upon request.

## Supplementary Materials

The following supporting information can be downloaded at: https://www.mdpi.com/article/10.3390/bioengineering9060248/s1, Figure S1: Histopathological analysis of ankle joint. (A) No CAIA (NC) control ankle joint showed subchondral bone (Sb), bone marrow cells (Bm), and an absence of inflammation. (B) Ankle joint in CAIA mice showed hyperplastic synovium (Sh), extensive inflammation (In), and pannus formation (Pn). (C) Treatment of the CAIA mice with P + 27pL showed a lack of hyperplastic synovium (Sh), inflammation (In), pannus formation (Pn), or cartilage erosion (Ce). (D) Treatment of CAIA mice with P showed an absence of Pannus formation (Pn) or inflammation. (E) Treatment of CAIA mice with 27pL showed hyperplastic synovium (Sh), inflammation (In), pannus formation (Pn), and cartilage erosion (Ce). (F) Total lesion score measurement indicated increased inflammatory lesion in the CAIA mice. Treatment with prednisone showed a significant reduction in the lesion score of ankle joints. Results represent mean ± SEM. A one-way analysis of variance (ANOVA) followed by Tukey’s multiple comparison t-test was used to calculate the statistical difference. The Mann–Whitney non-parametric test was used to calculate the statistical difference between P and P + 27pL (*** *p* < 0.001). Figure S2: Histopathological analysis of articular cartilage erosion of ankle joint. (A) Histological analysis of the no CAIA (NC) control knee joint stained with Safranin O showed normal uncalcified cartilage (Uc), calcified cartilage (CC), and subchondral bone (Sb). (B) Ankle joints in CAIA mice showed cartilage degradation extending up to the Sb and CC. (C) CAIA mice treated with P + 27pL lacked cartilage degradation or CC. (D) Treatment of CAIA mice with P showed non-fibrillation of the articular cartilage. (E) 27pL was unable to stop the degradation and formation of calcified cartilage in CAIA mice. (F) Total lesion scores indicated increased inflammatory lesion in the CAIA mice. Treatment with P showed a significant reduction in the cartilage degradation lesion score. Results represent mean ± SEM. A one-way analysis of variance (ANOVA) followed by Tukey’s multiple comparison t-test was used to calculate the statistical difference. The Mann–Whitney non-parametric test was used to calculate the statistical difference between P and P + 27pL (*** *p* < 0.001).
