# Supplementary material for: Enhancing Prednisone-Based Arthritis Therapy with Targeted IL-27 Gene Delivery
Source: Bioengineering (Basel). 2022 Jun 9;9(6):248. doi: 10.3390/bioengineering9060248 (PMC9220267; doi:10.3390/bioengineering9060248)
Supplement: Supplementary file 1 [file bioengineering-09-00248-s001.zip › bioengineering-1728135-supplementary.pdf]

Supplementary Figures

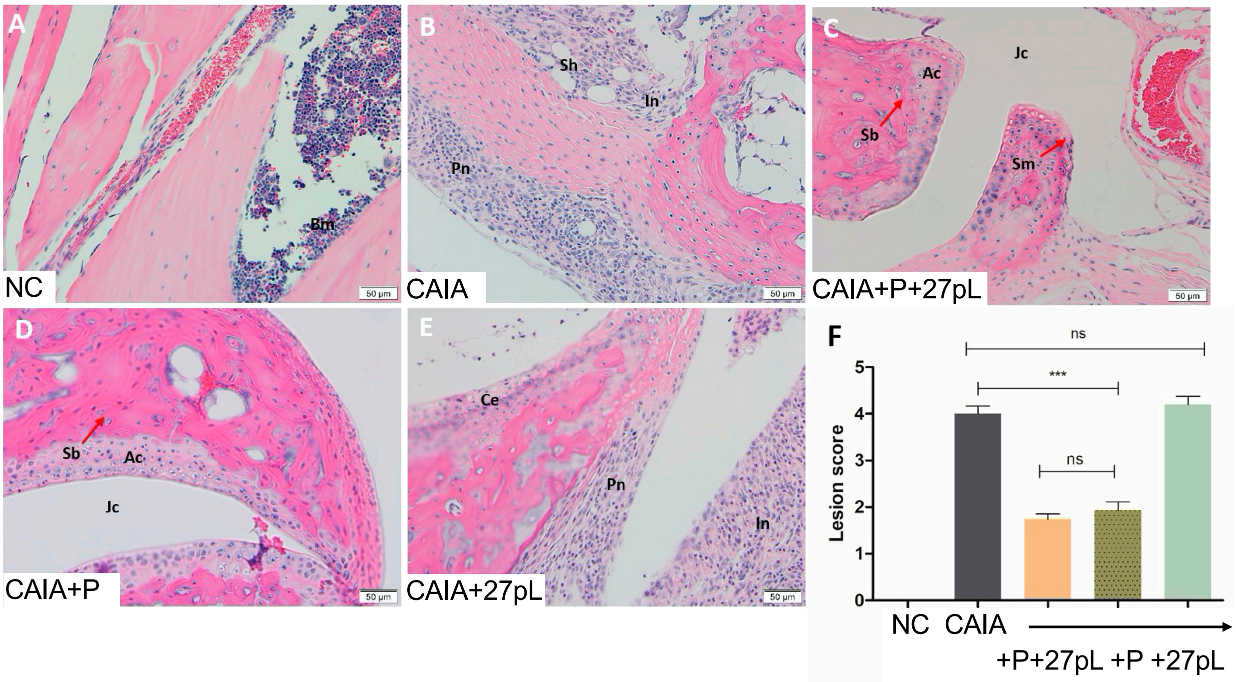

Figure S1: Histopathological analysis of ankle joint. (A) No CAIA (NC) control ankle joint showed subchondral bone (Sb), bone marrow cells (Bm), and an absence of inflammation. (B) Ankle joint in CAIA mice showed hyperplastic synovium (Sh), extensive inflammation (In), and pannus formation (Pn). (C) Treatment of the CAIA mice with P + 27pL showed a lack of hyperplastic synovium (Sh), inflammation (In), pannus formation (Pn), or cartilage erosion (Ce). (D) Treatment of CAIA mice with P showed an absence of Pannus formation (Pn) or inflammation. (E) Treatment of CAIA mice with 27pL showed hyperplastic synovium (Sh), inflammation (In), pannus formation (Pn) ,and cartilage erosion (Ce). (F) Total lesion score measurement indicated increased inflammatory lesion in the CAIA mice. Treatment with prednisone showed a significant reduction in the lesion score of ankle joints. Results represent mean ± SEM. A one-way analysis of variance (ANOVA) followed by Tukey's multiple comparison t-test was used to calculate the statistical difference. The Mann–Whitney non-parametric test was used to calculate the statistical difference between P and P + 27pL (*** $p < 0.001$).

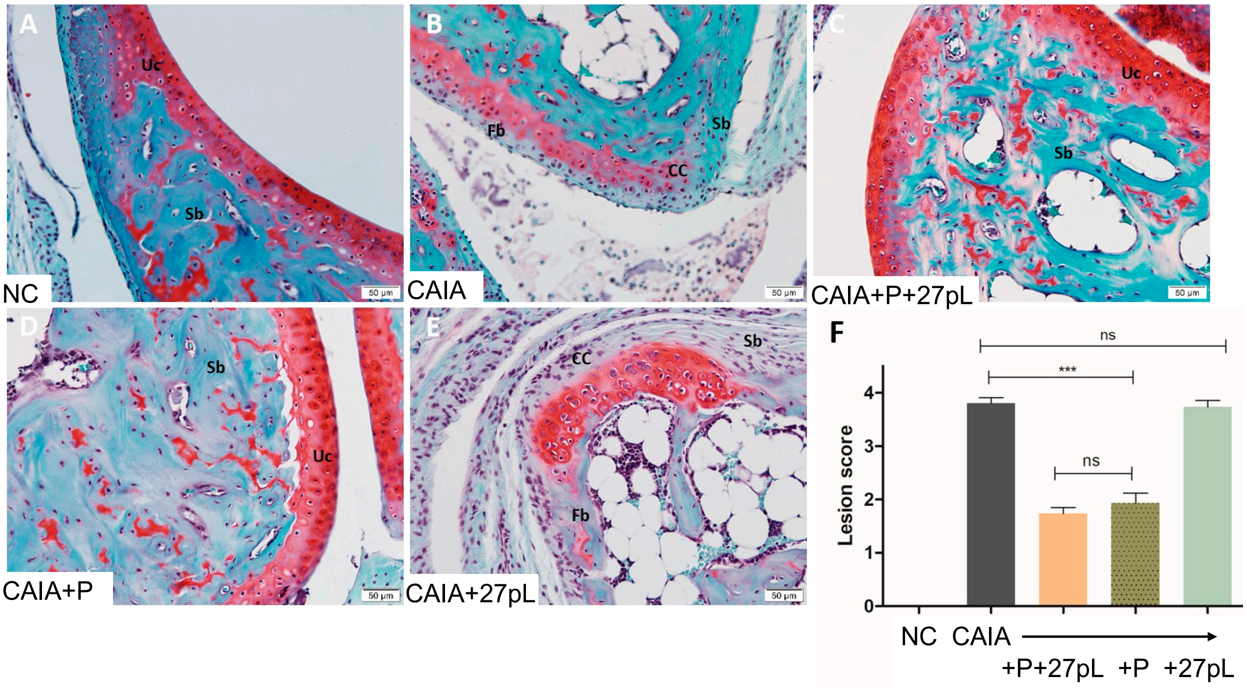

Figure S2: Histopathological analysis of articular cartilage erosion of ankle joint. (A) Histological analysis of the no CAIA (NC) control knee joint stained with Safranin O showed normal uncalcified cartilage (Uc), calcified cartilage (CC), and subchondral bone (Sb). (B) Ankle joints in CAIA mice showed cartilage degradation extending up to the Sb and CC. (C) CAIA mice treated with P + 27pL lacked cartilage degradation or CC. (D) Treatment of CAIA mice with P showed non-fibrillation of the articular cartilage. (E) 27pL was unable to stop the degradation and formation of calcified cartilage in CAIA mice. (F) Total lesion scores indicated increased inflammatory lesion in the CAIA mice. Treatment with P showed a significant reduction in the cartilage degradation lesion score. Results represent mean ± SEM. A one-way analysis of variance (ANOVA) followed by Tukey's multiple comparison t-test was used to calculate the statistical difference. The Mann–Whitney non-parametric test was used to calculate the statistical difference between P and P + 27pL (*** $p < 0.001$).
